# Preclinical Bioassay of a Polypropylene Mesh for Hernia Repair Pretreated with Antibacterial Solutions of Chlorhexidine and Allicin: An *In Vivo* Study

**DOI:** 10.1371/journal.pone.0142768

**Published:** 2015-11-10

**Authors:** Bárbara Pérez-Köhler, Francisca García-Moreno, Thierry Brune, Gemma Pascual, Juan Manuel Bellón

**Affiliations:** 1 Department of Surgery, Medical and Social Sciences, Faculty of Medicine and Health Sciences, University of Alcalá. Madrid, Spain; 2 Covidien – Sofradim Production, Trévoux, France; 3 Department of Medicine and Medical Specialties, Faculty of Medicine and Health Sciences, University of Alcalá. Madrid, Spain; 4 Networking Research Center on Bioengineering, Biomaterials and Nanomedicine (CIBER-BBN), Madrid, Spain; National University of Ireland, Galway (NUI Galway), IRELAND

## Abstract

**Introduction:**

Prosthetic mesh infection constitutes one of the major complications following hernia repair. Antimicrobial, non-antibiotic biomaterials have the potential to reduce bacterial adhesion to the mesh surface and adjacent tissues while avoiding the development of novel antibiotic resistance. This study assesses the efficacy of presoaking reticular polypropylene meshes in chlorhexidine or a chlorhexidine and allicin combination (a natural antibacterial agent) for preventing bacterial infection in a short-time hernia-repair rabbit model.

**Methods:**

Partial hernia defects (5 x 2 cm) were created on the lateral right side of the abdominal wall of New Zealand White rabbits (n = 21). The defects were inoculated with 0.5 mL of a 10^6^ CFU/mL *Staphylococcus aureus* ATCC25923 strain and repaired with a DualMesh Plus antimicrobial mesh or a Surgipro mesh presoaked in either chlorhexidine (0.05%) or allicin-chlorhexidine (900 μg/mL-0.05%). Fourteen days post-implant, mesh contraction was measured and tissue specimens were harvested to evaluate bacterial adhesion to the implant surface (via sonication, *S*. *aureus* immunolabeling), host-tissue incorporation (via staining, scanning electron microscopy) and macrophage response (via RAM-11 immunolabeling).

**Results:**

The polypropylene mesh showed improved tissue integration relative to the DualMesh Plus. Both the DualMesh Plus and the chlorhexidine-soaked polypropylene meshes exhibited high bacterial clearance, with the latter material showing lower bacterial yields. The implants from the allicin-chlorhexidine group displayed a neoformed tissue containing differently sized abscesses and living bacteria, as well as a diminished macrophage response. The allicin-chlorhexidine coated implants exhibited the highest contraction.

**Conclusions:**

The presoaking of reticular polypropylene materials with a low concentration of chlorhexidine provides the mesh with antibacterial activity without disrupting tissue integration. Due to the similarities found with the antimicrobial DualMesh Plus material, the chlorhexidine concentration tested could be utilized as a prophylactic treatment to resist infection by prosthetic mesh during hernia repair.

## Introduction

The surgical procedures developed to repair abdominal wall pathologies such as hernias have evolved over the last decades [1]. Traditional methods, such as autoplasty and suture repair, have been gradually replaced by prosthetic implants that achieve a significant reduction in the incidence of hernia recurrence [2,3]. Nevertheless, the use of biomaterials to repair hernia defects are associated with some post-surgical complications, with prosthetic mesh infection presenting one of the most devastating effects on patient health and also on the healthcare system [4].

Prosthetic mesh infection rate following hernia repair varies with the surgical procedure. Incisional hernias have infection rates of approximately 3.6% when repaired with laparoscopic surgeries [5] and approximately 10% following open surgeries [6]. Regardless of the anatomical location of the defect and the type of repair applied, the bacterial contamination of an implanted prosthetic device disrupts natural tissue repair and remodeling processes [7], which in turn jeopardizes the integrity of the implant and increases the risk of hernia recurrence [8].

Bacteria can enter the wound from the patient’s and/or the clinicians’ skin, from surgical instruments, or from the environment; this bacterial entry can then trigger a surgical-site infection (SSI) [9]. Once inside the body, bacteria interact with the surface of the implanted material through reversible physicochemical processes and consequently colonize the implant in an irreversible manner due to a strong adhesion to the implanted material and tissue mediated by the bacterial adhesins [10,11]. The initial mechanisms of bacterial adherence to the surface of the implanted biomaterial are thus a key step in the pathogenesis of the infection. *Staphylococcus aureus* (*Sa*) and *S*. *epidermidis* (*Se*) represent the two major sources of SSI and prosthetic mesh infections [12].

One of the main consequences of bacterial adherence to the mesh surface is the development of biofilms. The interactions among bacteria and their production of an exopolysaccharide capsule promotes the development of complex bacterial communities characterized by a potent adhesion to the substrate and an increased resistance to the action of drugs and host immune cells [13–15] Moreover, the presence of foreign bodies such as implanted biomaterials and surgical sutures decreases the bacterial load required to promote a SSI by approximately two thirds [16].

In an attempt to reduce the risk of infection after hernia repair, preoperative prophylaxis through the systemic administration of antibiotics [17] or the impregnation of the prosthesis in antibiotic solutions prior to the implantation [18] are commonly practiced in clinical settings and are highly recommended in high-risk patients undergoing surgery [18]. Nonetheless, in some circumstances the antibiotic prophylaxis is controversial, particularly in surgical procedures for inguinal hernia repair [19,20], since this strategy cannot guarantee absolute effectiveness [21,22]. Furthermore, the extended or inadequate use of antibiotic prophylaxis can lead to several adverse effects [23] and promote the occurrence of novel antibiotic resistances [24], so the local administration of antiseptic solutions is becoming more frequent. The utilization of antibacterial agents or antiseptics could avoid the development of novel resistances [25] and their local/topical application could reduce the systemic toxicity caused by antibiotic treatment [26].

One of the most widely utilized antiseptics is chlorhexidine (CHX). This is a potent antibacterial agent with proven activity against gram-positive, gram-negative bacteria and yeasts [27] which is frequently used for skin disinfection [28] and soaking of surgical devices such as vascular catheters [29]. At physiological pH, the positive charges of CHX destabilize the bacterial wall, altering the osmotic equilibrium and inducing the bacterial death [27].

Molecules of natural origin can also exhibit antibacterial activity. Historically, garlic and its derivatives have caused strong interest due to their antibacterial, antifungal and antioxidant properties [30]. Garlic cloves contain alliin, a sulfoxide compound that reacts with the enzyme alliinase (alliin lyase) when cloves are crushed, producing allicin [31]. Allicin is an oxygenated sulphur compound which exerts antibacterial activity mediated by chemical interactions with the sulfhydryl compounds of bacterial enzymes, provoking their inactivation [32]. This antibacterial agent has activity against a wide broad of gram-positive, gram-negative bacteria [30] and resistant strains such as methicillin resistant *S*. *aureus* (MRSA) [31].

In a recent *in vitro* study [33], our group demonstrated that the presoaking of reticular polypropylene (PP) meshes with a low concentration of CHX and a combination of CHX with allicin efficiently inhibited the adhesion of *Sa* to the material surface. Given the positive results obtained, the aim of the present work is to evaluate *in vivo* in a rabbit model of hernia repair the effectiveness of presoaking PP reticular meshes with these two antiseptics to combat *Sa*-contamination. The performances of these meshes were compared with the behavior of a commercially available laminar expanded polytetrafluoroethylene (ePTFE) mesh that exerts antibacterial activity.

## Material and Methods

### Preparation of the bacterial inocula

The bacterial strain *Sa* ATCC25923 (subspecies *aureus* Rosenbach 1884; CECT, Valencia, Spain) was utilized. A cryovial containing *Sa* was thawed, plated on Lysogeny Broth (LB) agar plates and incubated for 24 h at 37°C. A single colony was inoculated into 25 mL of LB medium and kept overnight at 37°C. The absorbance (OD600) was read by spectrophotometry and adjusted with sterile 0.9% saline to an OD600 equivalent to approximately 1 x 10^8^ CFU/mL. Two ten-fold serial dilutions were performed to generate a 10^6^ CFU/mL inoculum. The number of viable bacteria in the inoculum was determined with the spot plaque method in triplicate. Using the 10^6^ CFU/mL inoculum, five ten-fold serial dilutions were prepared and 100 μL of each dilution plated on LB agar plates. After 24 h of incubation at 37°C, the plates were counted and the viable CFU/mL were determined using the following calculation: CFU/mL = number of CFU x dilution factor (10x) / volume plated (0.1 mL).

### Antibacterial solutions

The following sterile antimicrobial solutions were utilized:

- CHX: Solution of chlorhexidine digluconate (Santa Cruz Biotechnology, Texas, USA) diluted to 0.05% in ultrapure water.
- Allicin-CHX: Mixed solution containing 900 μg/mL allicin (Allimed Liquid; Allicin International Ltd., Stratford, UK) and 0.05% CHX.

### Agar well diffusion test

The efficacy of the antibacterial solutions was tested *in vitro* by means of an agar well diffusion test. Using sterile swabs, 1 mL of the *Sa* 10^6^ CFU/mL inoculum was utilized to spread bacterial lawns onto 15 LB agar plates. Circular wells (8 mm in diameter, 4 mm in depth) were then punched in the middle of each contaminated plate and filled with 100 μL of the corresponding CHX or allicin-CHX solution (n = 5 plates per group), and plates filled with sterile 0.9% saline were used as control (n = 5). After 24 h of incubation at 37°C, the zones of inhibition were recorded by measuring two perpendicular diameters per plate. The results were expressed as the mean inhibition zone (mm) per treatment.

### Prosthetic materials and study groups

The following hernia repair prosthetic materials were utilized:

- Gore DualMesh Plus mesh (DM+) (W.L. Gore & Associates, Delaware, USA). Two-surface laminar antimicrobial ePTFE mesh coated with silver carbonate and chlorhexidine diacetate with thicknesses of 1.5 mm.
- Surgipro mesh (PP) (Covidien, Dublin, Ireland). Heavyweight, monofilamental, non-absorbable PP mesh (85 g/m^2^) with a pore surface area of 0.26 ± 0.03 mm^2^.

Immediately prior to the implantation, the PP materials were soaked in the different antibacterial solutions for 1 min, and the following study groups were established:

- DM+ (n = 7)
- PP + CHX (n = 7)
- PP + allicin-CHX (n = 7)

### Experimental animals

The study protocol adhered to the Animals in Research: Reporting *In Vivo* Experiments (ARRIVE) guidelines for the publication of animal studies [34]. The experimental animals used were 21 male New Zealand White rabbits weighing approximately 3,000 g, which were randomized into the different study groups. The study was carried out in strict accordance with the Guide for the Care and Use of Laboratory Animals of the National and European Institutes of Health (Spanish law 32/2007, Spanish Royal Decree 1201/2005, European Directive 2010/63/UE and European Convention of the Council of Europe ETS123). The study protocol was approved by the Committee on the Ethics of Animal Experiments of the University of Alcalá (registered code: CEI2013004). All procedures were performed at the Animal Research Center of Alcalá University.

### Surgical techniques and experimental design

To minimize pain, the animals were administered 0.05 mg/kg buprenorphine (Buprecare, Divasa Farmavic, Spain) 1 h before and 3 days after the surgical procedure. The animals were anesthetized using a mixture of ketamine hydrochloride (Ketolar; Parke-Davis, Spain) (70 mg/kg), diazepam (Valium; Roche, Spain) (1.5 mg/kg) and chlorpromazine (Largactil; Rhône-Poulenc, Spain) (1.5 mg/kg), which was administered intramuscularly 15 min before the surgery. In some cases, an additional dose of anesthetic was injected directly in the abdominal cavity during the course of surgery.

**Fig 1:**
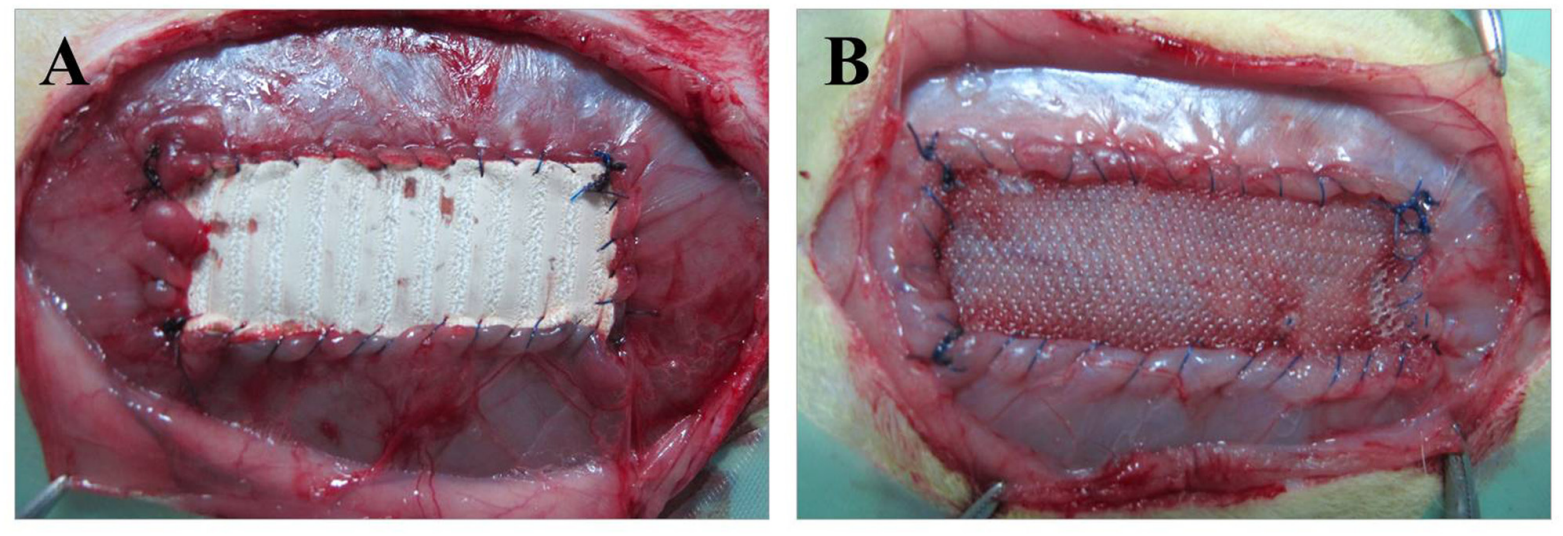
Surgical technique. Macroscopic image of the biomaterials after implant into the experimental animal. (A) DM+ implant, (B) PP implant presoaked in either CHX or allicin-CHX solutions.

Using a sterile technique, an approximately 5-cm longitudinal laparotomy was introduced on the right side of the midline. After dissecting the subcutaneous tissue, a 5 x 2 cm musculofascial defect was created at a 1-cm distance from the midline comprising the fascia and oblique muscles while sparing the transverse muscle and peritoneum. The defect was then inoculated with 0.5 mL of the *Sa* 10^6^ CFU/mL suspension previously prepared, and the corresponding biomaterial was fixed to the margins of the defect by running a PP 4/0 suture interrupted only at the implant corners (Fig 1). The skin was closed over the implant by running a PP 3/0 suture. A total of 7 implants per study group were developed.

The animals were weighed daily to determine any weight loss or increase and were visually monitored for signs of surgical incision dehiscence, fistula or seroma formation, SSI and/or other complications. After 14 post-operative days, the animals were sedated with up to 20 mg/kg of xylazine (Rompun; Bayer, Leverkusen, Germany) and then euthanized in a CO_2_ chamber with increasing concentrations of CO_2_.

### Mesh contraction

Contraction of the meshes at 14 days post-implantation was determined by image analysis of photographs taken at the moment of the euthanasia. The photographs were scaled and the surface area of each mesh was measured using image analysis software (Image J, NIH; http://imagej.nih.gov/ij/). The area recorded for each implant was compared to the initial area of the meshes (5 x 2 cm). The results were expressed as the mean percentage of mesh contraction suffered by each study group.

### Macroscopic observations

**Fig 2:**
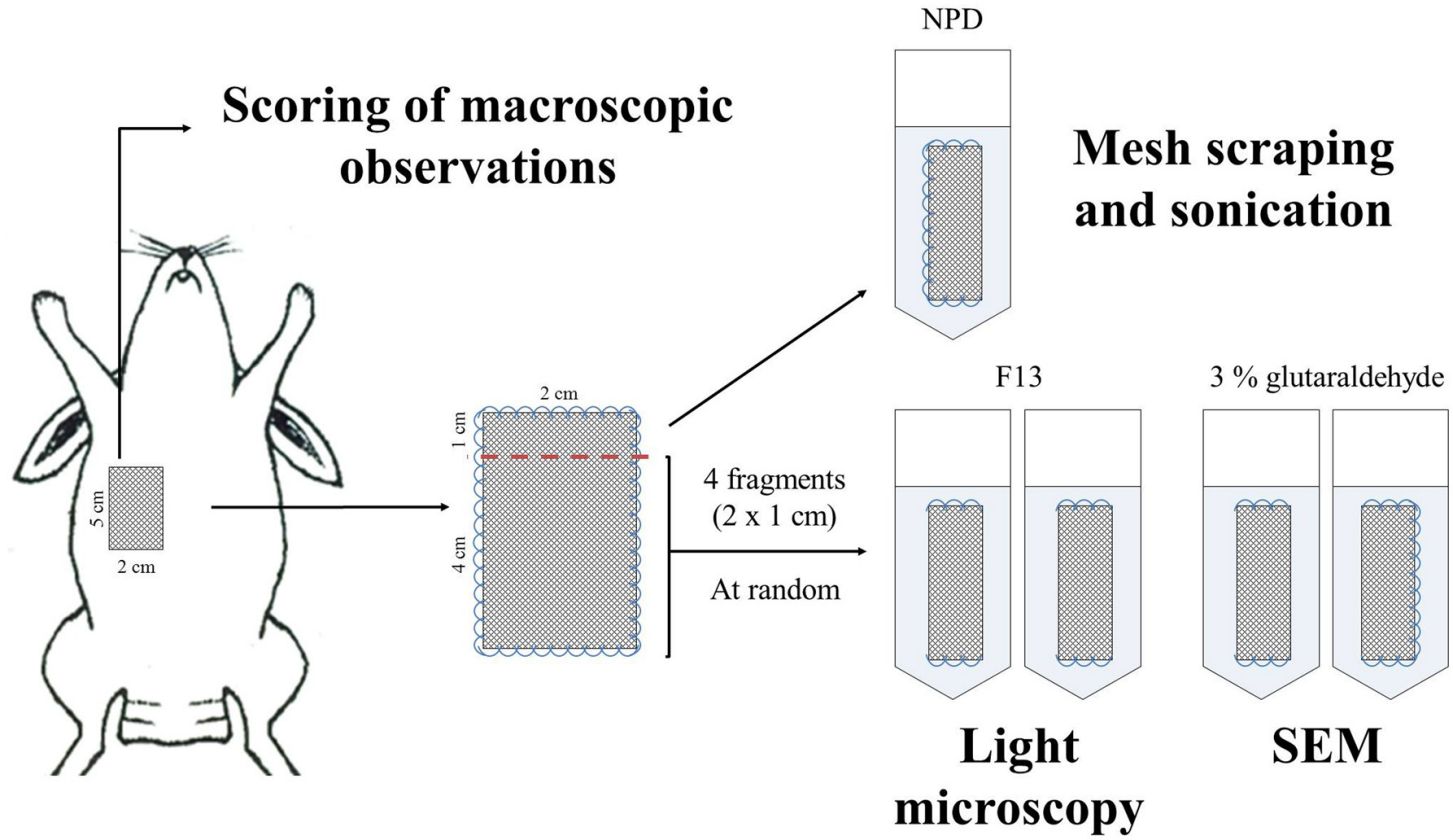
Experimental design. Diagram showing the harvesting and processing of the tissue samples to carry out the macroscopic, microbiological and histological evaluation of the different implants.

Following euthanasia, the implants were examined to score the most relevant macroscopic observations for each specimen using the scoring system shown in Table 1, specifically designed for this purpose. If seroma formation was detected, the liquid was aspirated with a sterile syringe, stored at 4°C until the end of the intervention, and 100 μL of each sample was plated on LB agar plates and incubated at 37°C for 24 h to determine the presence of free-floating bacteria in the seroma. After monitoring the implants, meshes plus surrounding host tissue were harvested from the interfaces of the prosthesis/visceral peritoneum and the prosthesis/subcutaneous tissue for microbiological, morphological and immunohistochemical studies, according to the diagram shown in Fig 2.

**Table 1:** Scoring system utilized to evaluate the implants at the moment of the euthanasia.

**Infection assessment—Main features**					
**Skin necrosis**	Absence		Presence		
**Fistula**	Absence		Presence		
**Swelling/Edema**	Absence	Soft Moderate area	Soft Large area	Hard Moderate area	Hard Large area
**Purulent material**	Absence	A few sites along the suture line	All around the implant	Covering < 50% of surface	Covering > 50% of surface
**Infection assessment—Secondary features**					
**Vascularization**	Normal	Moderate		Severe	
**Thrombosis**	Absence	Moderate		Severe	
**Tissue integration assessment**					
**Encapsulation**	Absence	Moderate		Severe	
**Tissue integration**	Complete	Partial integration		Delamination	

### Efficacy of the antimicrobial coatings

To evaluate the antibacterial effectiveness of the DM+ and the PP material soaked in CHX or allicin-CHX, mesh fragments with dimensions of approximately 2 x 1 cm were harvested and individually transferred to glass tubes containing 20 mL of sterile Neutralizing Pharmacopoeia Diluent (NPD) (8.5 g NaCl, 2.5 mL Tween-80, 0.35 g lecithin, 997.5 mL distilled water). Under sterile conditions, the mesh was separated from the surrounding tissue and both sides of the material were scrapped with a scalpel blade. The glass tubes containing the NPD, explanted mesh and remaining tissue were subjected to a 10-min sonication pulse at 40 kHz using a Bransonic 3800-CPXH ultrasonic cleaning bath (Branson Ultrasonics, Connecticut, USA). The tubes were thoroughly vortexed for 1 min and the supernatant was used to perform five 1/10 serial dilutions. A volume of 100 μL of each dilution was seeded onto LB agar plates and incubated for 24 h at 37°C. The plates were counted and the viable CFU per mesh fragment were determined using the following formula: Viable CFU per mesh fragment = viable CFU/mL x 20 mL (volume of NPD in the tube). Using these data, the bacterial clearance was calculated as the percentage of animals with sterile cultures in each of the different study groups.

### Morphological analyses

For light microscopy, the tissue specimens were fixed in F13 solution (60% ethanol, 20% methanol, 7% polyethylene glycol, and 13% distilled water) and embedded in paraffin. Tissue sections (5 μm thickness) were sliced, stained with hematoxylin-eosin and Masson’s trichrome (Goldner-Gabe) and examined under a Zeiss Axiophot light microscope (Carl Zeiss, Oberkochen, Germany). For scanning electron microscopy (SEM), the tissue specimens were fixed in 3% glutaraldehyde, placed in Millonig buffer (pH 7.3) and dehydrated in a graded ethanol series (30%, 50%, 70%, 90%, and 100%, each incubated for 15 min). The critical point was reached in an E-3000 Polaron instrument (Polaron Ltd., Newhaven, UK). The pieces were metalized with gold palladium and visualized with a Zeiss DSM950 scanning electron microscope (Carl Zeiss).

### Immunohistochemistry

Paraffin-embedded tissue sections were utilized to evaluate the bacterial colonization of the mesh, the surrounding tissue, and the macrophage activity. The tissue sections were de-paraffinized in xylene and dehydrated in a graded ethanol series (100%, 96%, and 70%, each incubated for 5 min), hydrated and equilibrated in Tris-buffered saline (TBS; pH 7.4). Non-specific protein interactions were blocked through incubation with 3% bovine serum albumin (BSA) for 30 min at room temperature. The sections were incubated with the monoclonal antibodies against *Sa* (ab37644; Abcam, Cambridge, UK) and rabbit macrophages RAM-11 (M-633; Dako, Glostrup, Denmark) in the alkaline phosphatase-labeled avidin-biotin procedure. The method included the following steps: incubation with primary antibody (1:500 for anti-*Sa* and 1:50 for anti-RAM-11, in TBS) for 60 min; incubation with immunoglobulin G (IgG) and biotin (1:1,000 in TBS) for 45 min; and labeling with streptavidin alkaline phosphatase (1:200 in TBS) for 60 min. These steps were conducted at room temperature. Negative controls were subjected to 3% BSA instead of the primary antibodies. The images were revealed using a chromogenic substrate containing naphthol phosphate and fast red. Cell nuclei were counterstained for 1 min with acid hematoxylin. The presence of bacteria was qualitatively evaluated. Labeled macrophages were quantified by performing counts in 10 light microscopy fields (magnification x20) per tissue sample. A total of 70 fields per study group were counted, and the results were expressed as the percentage of positively stained cells out of the total number of cell nuclei per field.

### Statistical analyses

The data collected from the different experiments were represented as the mean ± standard error of the mean. Statistical analysis was performed using one-way analysis of variance (ANOVA) and Bonferroni as *post hoc* test. All the statistical analyses were performed using the GraphPad Prism 5 computer package (La Jolla, California, USA) for Windows. The significance level was set at p<0.05.

## Results

### *In vitro* study

**Fig 3:**
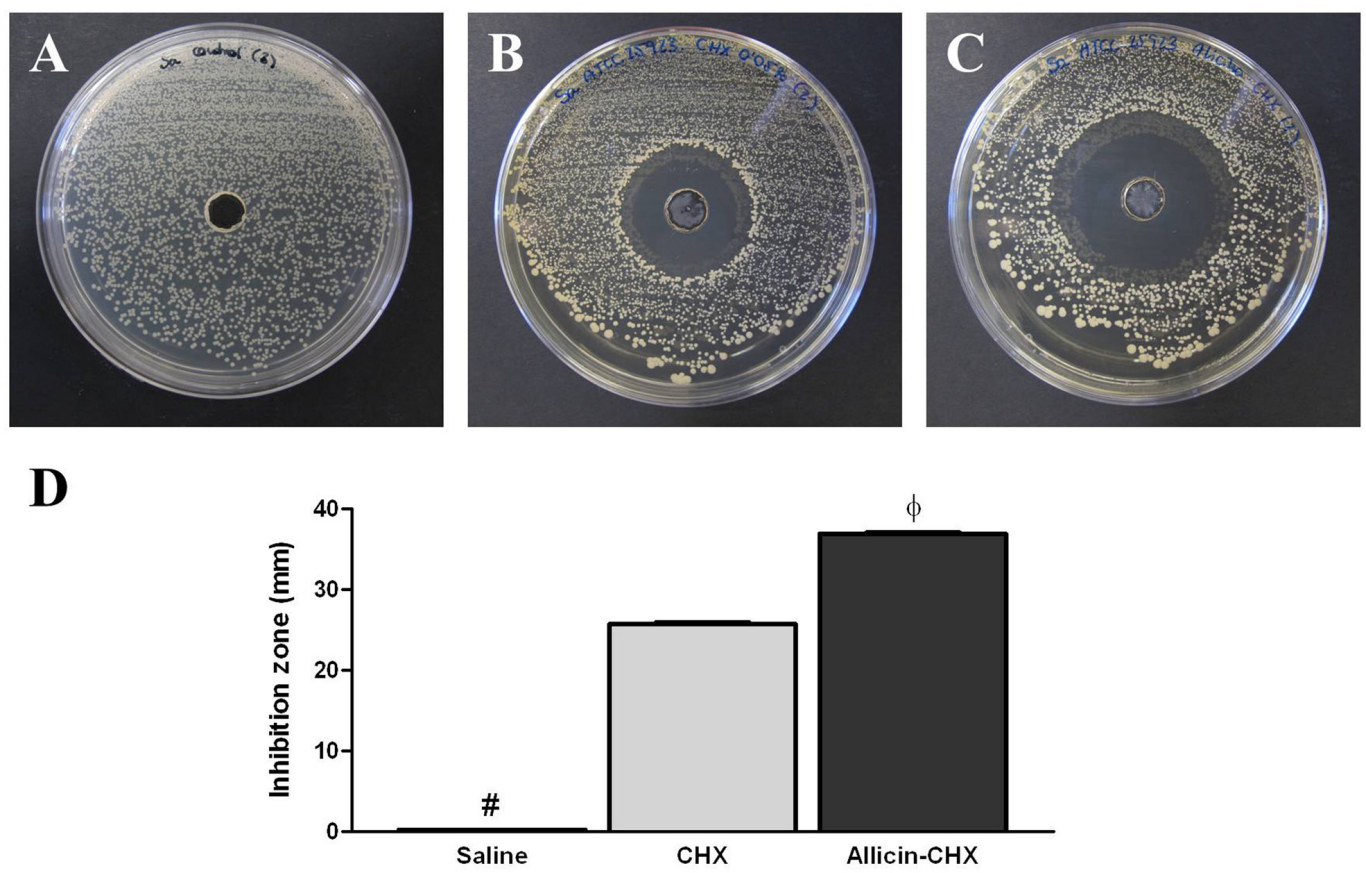
*In vitro* agar well diffusion test. Representative images of the inhibition zones after 24 h of incubation at 37°C by the (A) CHX and (B) allicin-CHX solutions utilized to soak the PP meshes. (C) Sterile saline used as control did not provoke bacterial growth inhibition. (D) Mean diameter of the inhibition zones (mm). The results are expressed as the mean ± standard error of the mean for 5 samples. The allicin-CHX solution provoked significantly wider inhibition halos than the CHX treatment. #: saline vs CHX and allicin-CHX (p<0.001); ф: CHX vs saline and allicin-CHX (p<0.001).

The *Sa* suspension used to inoculate the experimental animals and to determine the activity of the CHX and allicin-CHX solutions *in vitro* contained a mean viable bacteria level of 1.45 x 10^6^ CFU/mL. The agar well diffusion test confirmed the effectiveness of the two antibacterial treatments tested because both provoked the development of inhibition halos in all the *Sa*-pre-lawn agar plates after 24 h of the contamination, while all the plates containing saline were fully contaminated (Fig 3). The measure of the halo diameters revealed significantly wider inhibition zones created by the allicin-CHX solution (36.90 ± 0.19 mm) in comparison with CHX (25.70 ± 0.26 mm) (p<0.001).

### Postoperative follow-up

No mortality was observed in any of the study groups. Following the surgery, all of the animals exhibited no behavioral signs that might imply a decrease in welfare, regardless of the implanted mesh. There was no wound dehiscence, inflammation, fistula formation, skin erythema or necrosis. After 14 days of the implantation, the animals from the different study groups showed normal percentages of weight increase, with values of 1.06 ± 0.02% for DM+, 1.03 ± 0.02% for PP + CHX and 1.06 ± 0.01% for PP + allicin-CHX.

### Mesh contraction

The mesh contraction measured for the different study groups showed mean percentages of 6.38 ± 1.18% for DM+, 12.14 ± 2.33% for PP + CHX and 14.15 ± 1.88% for PP + allicin-CHX implants. While no significant differences were observed between the two coated PP study groups, the contraction of the PP + allicin-CHX implants was statistically higher than the recorded for DM+ (p<0.05).

### Morphological studies

**Fig 4:**
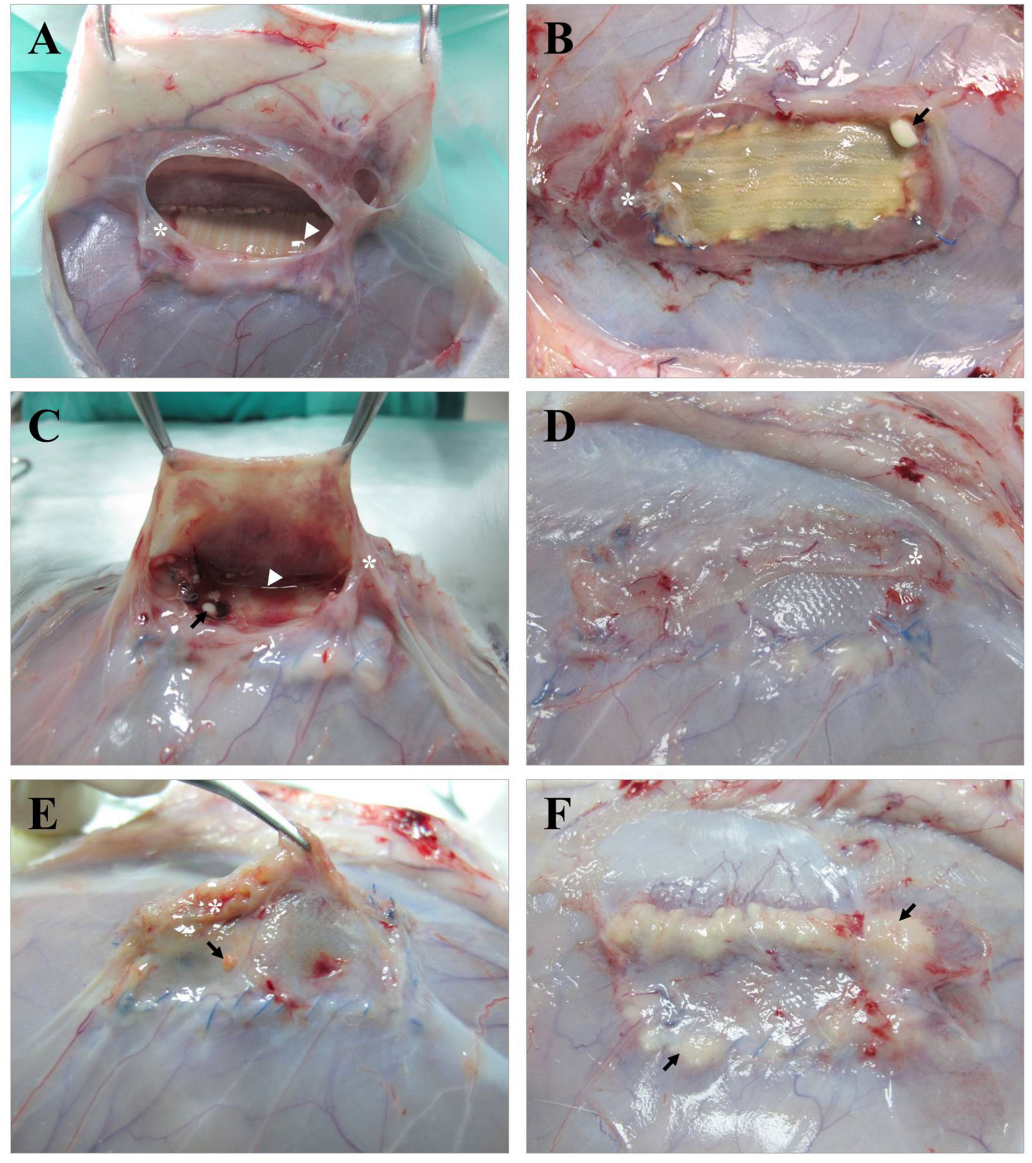
Macroscopic findings. Appearance of the different meshes after 14 days of implantation and *Sa* contamination. (A, B) DM+ implants showing thick fibrous encapsulation (*), seroma formation (▶) and the presence of dispersed purulent material (→) associated with the mesh anchorage, while the main body of the implant remains clean. (C, D) PP + CHX implants showing similar behavior to the previous ones, with the exceptions of a more intense vascularization and total mesh integration into the host tissue. (E, F) PP + allicin-CHX implants show large amounts of purulent material (→) covering different areas of the implant surface.

At necropsy, and prior to the sample harvesting, the wound area of the animals was subjected to a comprehensive macroscopic examination of the mesh and the surrounding tissues to evaluate the response of the implant to the bacterial infection (Table 1). The DM+ implants (Fig 4A and 4B) partially integrated in the host tissue were surrounded by a fibrous capsule containing transparent to semi-turbid exudate; four of the meshes showed small amounts of purulent material associated with the PP suture, and seroma was collected from 2 specimens and was refrigerated for further analysis. The PP + CHX implants (Fig 4C and 4D) were encapsulated, totally integrated into the host tissue and showed moderate vascularization over the mesh; as observed previously, the presence of small purulent material attached to the PP suture filament was detected, and turbid seroma were collected from 3 specimens. The observations of the PP + allicin-CHX implants (Fig 4E and 4F) were characterized by the presence of several amounts of purulent material covering the suture filament and scattered over areas of the mesh; the biomaterials were totally integrated in the host tissue and none of the specimens exhibited seroma formation.

The seromas collected from both the DM+ and the PP + CHX groups were plated on LB agar plates and incubated at 37°C. After 24 h of the incubation, none of the samples showed any colony growth, and the seromas were thus considered free of bacteria.

### Bacterial colonization on the implant surface

The results from the sonication demonstrated the great antibacterial activity of the DM+ implants with 86% of bacterial clearance because 6 of the samples were sterile and the last sample showed 4.90 x 10^7^ CFU per mesh fragment. The same bacterial clearance was recorded for the PP + CHX group; however, the only contaminated specimen in this group showed lower bacterial yields than the contaminated DM+ sample, with 1.40 x 10^3^ CFU per mesh fragment. The poorest behavior was recorded for the PP + allicin-CHX group, with 57% bacterial clearance and 3 contaminated specimens out of 7, with mean counts of 1.62 x 10^5^ CFU per mesh fragment. There were no statistical differences among the different study groups. Table 2 summarizes the bacterial counts of the samples yielded after the sonication.

**Table 2:** Viable CFU per mesh fragment (2 x 1 cm) of the *Sa*-contaminated implants.

Bacterial counts after sonication (CFU per mesh fragment)			
Sample	DM+	PP + CHX	PP + allicin-CHX
**Specimen 1**	0 (sterile)	0 (sterile)	7.60 x 10^3^
**Specimen 2**	0 (sterile)	1.40 x 10^3^	0 (sterile)
**Specimen 3**	0 (sterile)	0 (sterile)	0 (sterile)
**Specimen 4**	4.90 x 10^7^	0 (sterile)	0 (sterile)
**Specimen 5**	0 (sterile)	0 (sterile)	0 (sterile)
**Specimen 6**	0 (sterile)	0 (sterile)	4.76 x 10^5^
**Specimen 7**	0 (sterile)	0 (sterile)	3.40 x 10^3^

### Histology

**Fig 5:**
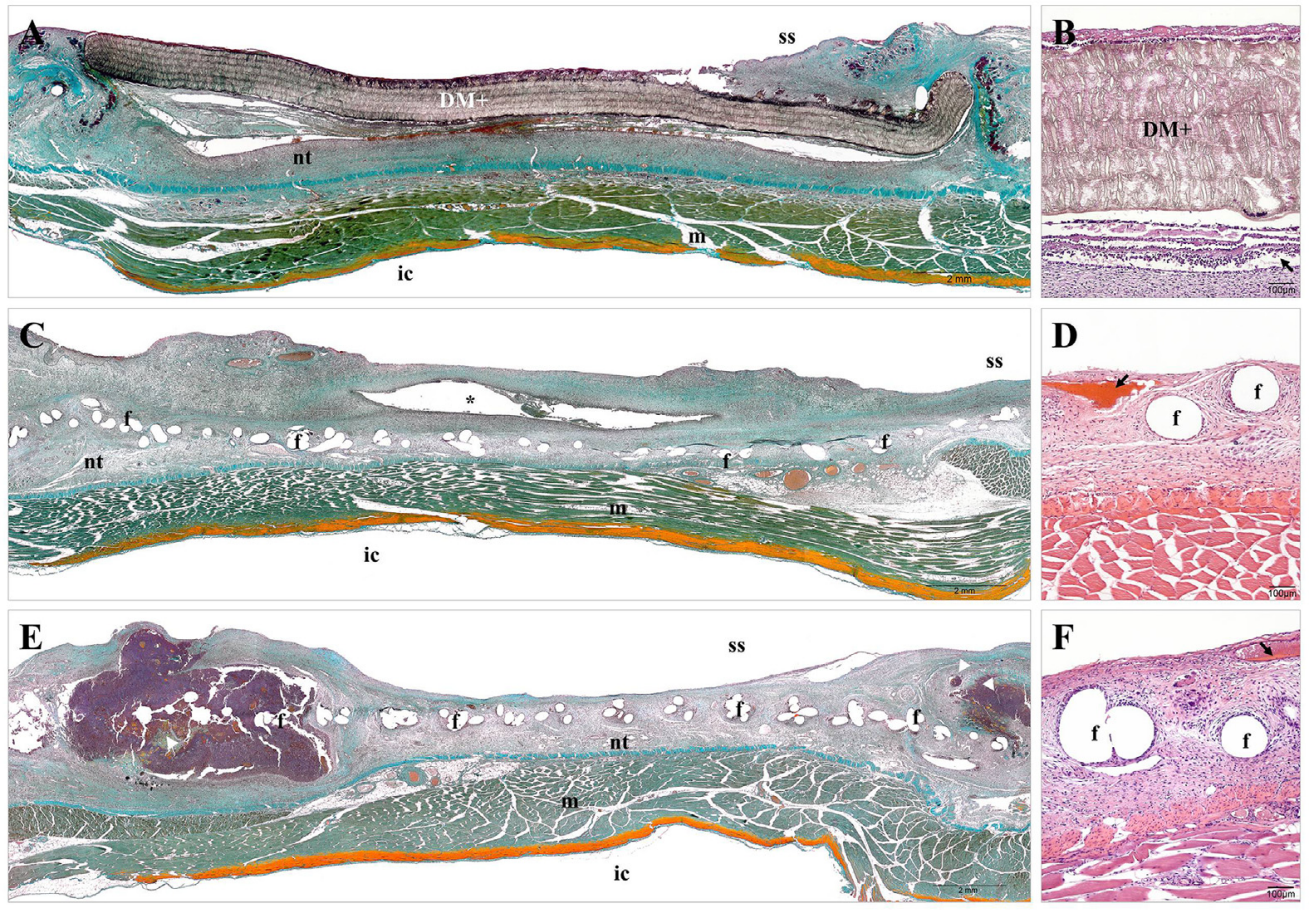
Tissue integration of the implanted biomaterials. Panoramic compositions (Masson´s trichrome staining, x50) and light microscopy (hematoxylin-eosin, x100) micrographs of the different study groups. (A, B) The DM+ implants were partially integrated in the host tissue and showed a dense neoformed connective tissue containing inflammatory cells and angiogenesis (→) below the mesh. (C, D) The PP + CHX implants were fully integrated and exhibited cavities (*) in the neoformed tissue containing non-drained seroma and showed loose connective tissue surrounding the mesh filaments with angiogenesis (→). The PP + allicin-CHX implants displayed loose connective tissue infiltrating the mesh with different-sized abscesses (▶) and angiogenesis (→). f: mesh filaments; ic: intraperitoneal cavity; m: muscle; nt: neoformed tissue; ss: subcutaneous side.

Consistent with the macroscopic outcomes, the histological evaluation of the tissue specimens evidenced that the DM+ implants (Fig 5A and 5B) were not fully integrated in the host tissue. These implants were surrounded by a layer of loose connective tissue, which grew denser in the areas of mesh anchorage. The neoformed tissue was slightly vascularized below the implant and presented a barrier of inflammatory cells along the material, keeping the mesh wall free of cell infiltration. The PP + CHX implants (Fig 5C and 5D) were completely integrated into the host tissue, showing loose connective tissue infiltrated in the mesh, with presence of inflammatory cells and extracellular matrix fibers surrounding the PP filaments in a concentric fashion. The vascularization of the neoformed tissue was evident, and dispersed cavities surrounded by granulocytic tissue were observed in the connective tissue of specimens developing seroma. The same tissue response was observed in the PP + allicin-CHX implants (Fig 5E and 5F); however, these implants exhibited different-size abscesses at areas of mesh anchorage, containing large amounts of inflammatory cells, cell debris and detritus.

**Fig 6:**
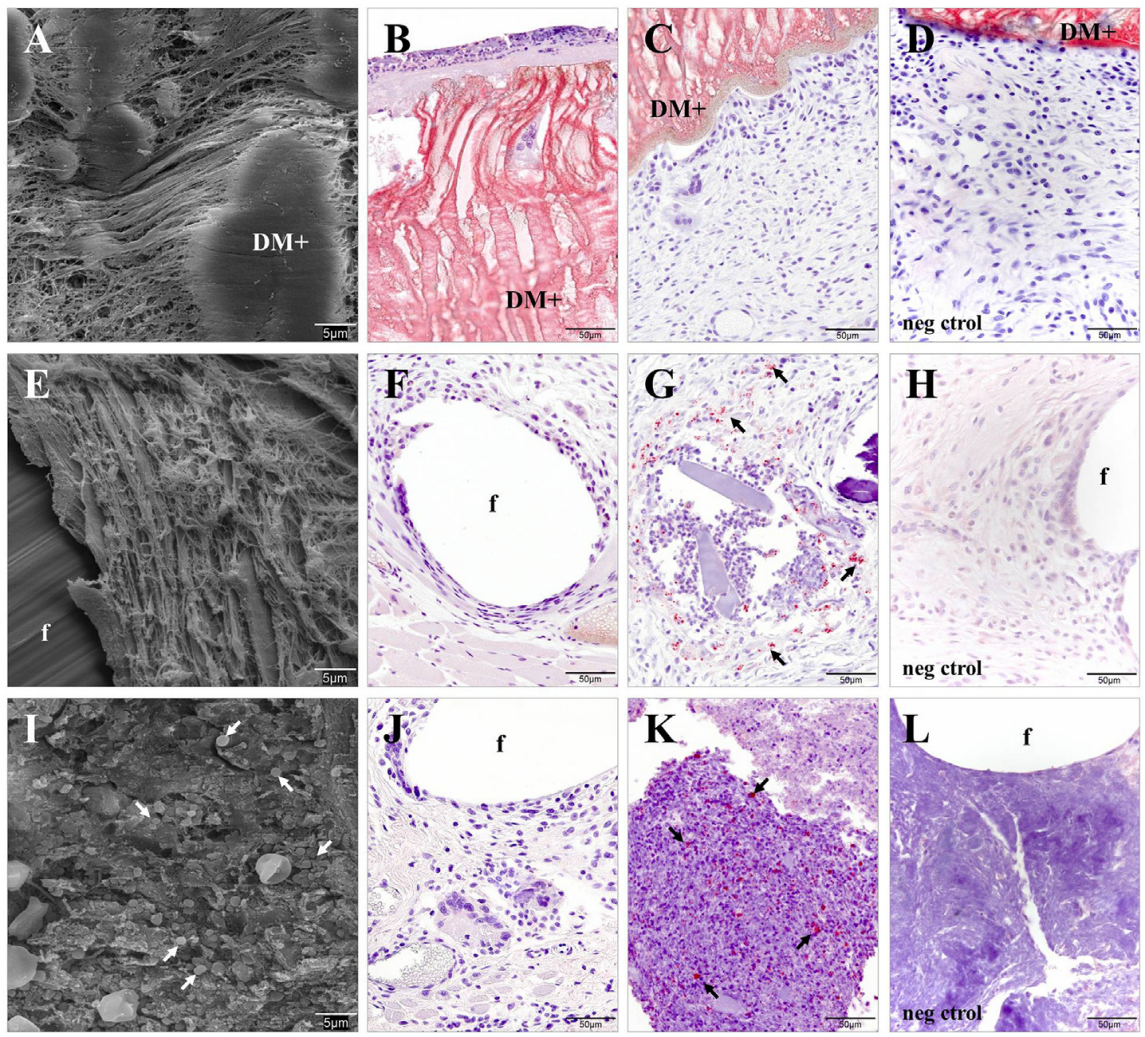
Bacterial adhesion to the implant surface. SEM micrographs (x2000) and *Sa* immunolabeling (x400) of the different study groups. (A-C) The DM+ implants showed no presence of bacteria in either the neoformed connective tissue or in the mesh surface. (E-G) The PP + CHX implants did not exhibit bacteria in the neoformed tissue, apart from the single exception of one specimen (F), which yielded bacteria (→) following mesh sonication. (I-K) The connective tissues of the PP + allicin-CHX implants were free of bacteria, although microorganisms (→) were found inside and surrounding the abscesses. The negative controls of the (D) DM+, (H) PP + CHX and (L) PP + allicin-CHX implants showed no immunostaining. f: mesh filaments.

The SEM and *Sa*-immunolabeling micrographs of the tissue specimens from DM+ implants showed no bacteria embedded in either the connective tissue or attached to the mesh surface (Fig 6A–6C), even in the animal that had produced positive bacterial yields after sonication. The PP + CHX implants (Fig 6E–6G) exhibited similar results, with the exception of a few bacteria located solely at the mesh fixation site of the only contaminated animal, as previously determined by sonication. In the PP + allicin-CHX group (Fig 6I–6K), bacteria were detected inside and surrounding the abscesses of the contaminated specimens with the neoformed tissue free of microorganisms.

### Macrophage response

**Fig 7:**
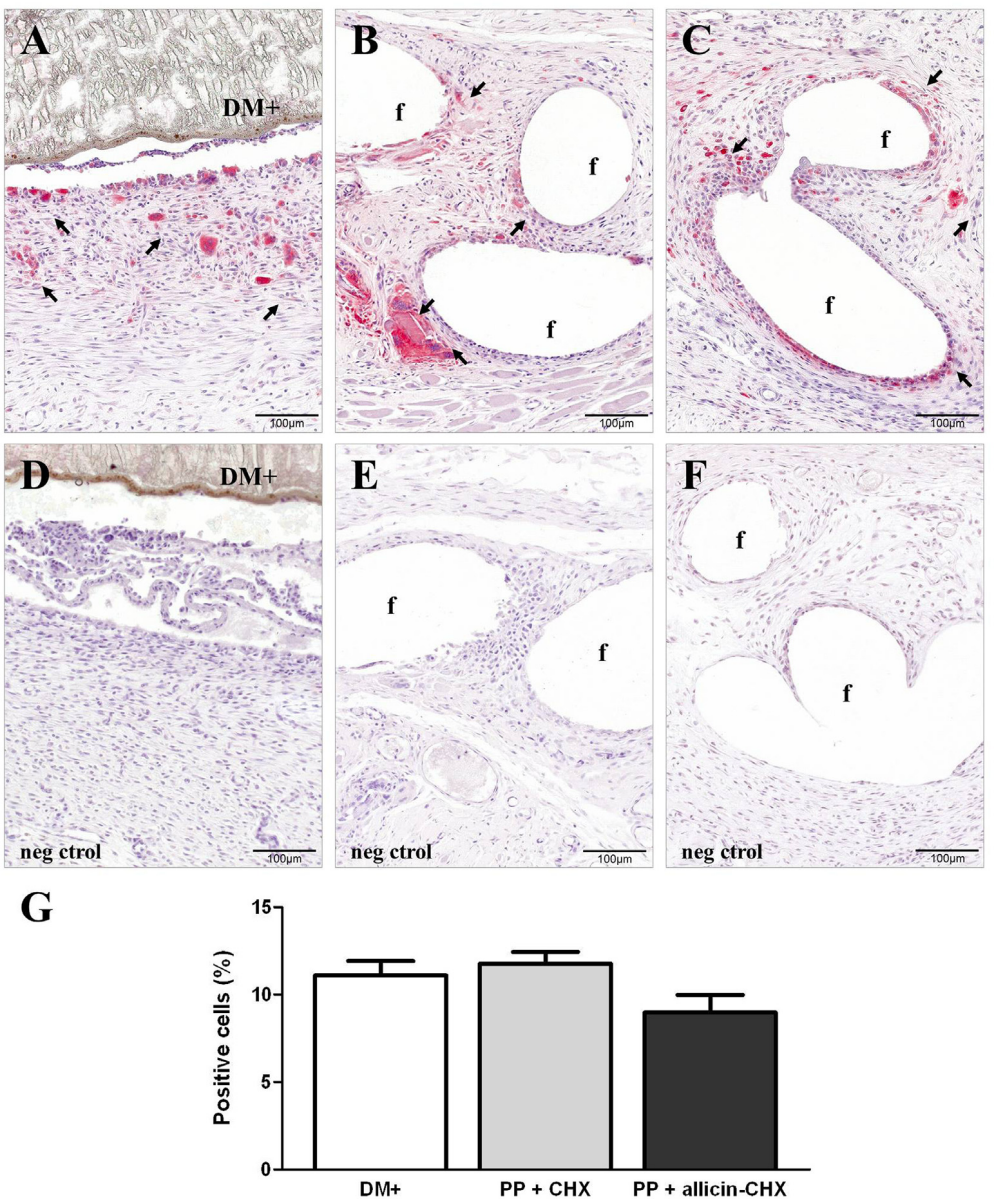
Foreign-body reaction. RAM-11 immunostaining (x200) of the (A) DM+, (B) PP + CHX, and (C) PP + allicin-CHX implants showing the presence of labeled macrophages (→) in the neoformed tissue. (D-F) Negative controls of the DM+ (D), PP + CHX (E) and PP + allicin-CHX (F) implants showing no immunostaining. (G) Positive cell percentages recorded after 14 days of implant. The results are expressed as the mean ± standard error of the mean for the total of micrographs counted (7 specimens per study group, 10 micrographs per specimen). The lower percentage of RAM-11 positive cells recorded for the PP + allicin-CHX implants was not statistically significant.

A moderate presence of cells labeled with the RAM-11 monoclonal antibody was observed in all the study groups, which were mainly found in areas of the connective tissue next to the biomaterials and the suture filaments. In the DM+ group (Fig 7A), the macrophages showed a tendency to form a cell barrier between the prostheses and the neoformed tissue without any evidence of cell infiltration into the mesh wall. The PP + CHX (Fig 7B) and the PP + allicin-CHX implants (Fig 7C) exhibited labeled macrophages and multinucleated giant foreign body cells surrounding the PP filaments. These cells have also infiltrated into the abscesses of the PP + allicin-CHX implants. The macrophage counts (Fig 7G) revealed a lower number of RAM-11 positive cells in the PP + allicin-CHX implants compared to both DM+ and PP + CHX implants, although there were no statistical differences among the different study groups.

## Discussion

Given the great impacts of prosthetic infection for healthcare, the design of biomaterials provided with antibacterial activity is essential. In this regard, coating meshes for hernia repair with antiseptics could be a favorable strategy to prevent prosthetic mesh infection, avoiding the bacterial adhesion to the material surface. Mesh soaking in antiseptics prior to implantation is an easy and fast procedure which could reduce the negative side effects associated with the extended use of antibiotics [25,26]. In the present work we have evaluated the short-time performance of two compounds with antibacterial activity to soak PP meshes in a rabbit model of *Sa* infection. If bacterial adhesion occurs, it will probably happen during the first hours post-surgery. If the treatments are not effective, the infection should be strongly consolidated after a few days. Supposing that infection occurs, a short-time study would give us important data regarding not only the distribution of the bacterial contamination along the implant but also the possible alterations of the tissue repair process. We considered thus that 14 days postoperatively was an adequate study time to evaluate both events.

Regardless of the presoaking solution, both PP implants exhibited better tissue integration than DM+. This observation was recorded after 14 post-operative days and therefore does not show the complete process of tissue repair which requires longer study times. However, this short-time behavior of the different implants is consistent with previous observations stating that the reticular materials allow the infiltration of the granulation tissue through the mesh pores, while the laminar ones usually favor implant encapsulation [35]. The DM+ and the PP + CHX implants showed high and similar bacterial clearance with only one animal per study group yielding bacteria after 14 days of contamination. However, the bacterial load of the respective animals differed, with the PP mesh contaminated animal exhibiting a bacterial load that was approximately 4-log values lower than that of the DM+. This result can be attributed to the fact that the reticular meshes have a much lower contact surface area where bacteria can adhere relative to the laminar materials [36]. Furthermore, the microporous architecture of the DM+ may hinder the arrival of host immune cells to the contaminated area [21,37].

When comparing the PP implants we observed differences depending on the solution used to presoak the materials. The presence of purulent material over the implant and abscesses embedded in the neoformed tissue, together with the lower bacterial clearance and the reduction in the percentage of RAM-11 labeled cells, suggests that the allicin is somehow disrupting the performance of the treatment. Some studies indicate that low concentrations of allicin and other garlic derivatives inhibit the synthesis of pro-inflammatory cytokines such as IL-1α, IL-1β, IL-12 and TNF-α [38,39] and other cytokines related to inflammatory processes such as IL-2, IL-6, IL-8 e IFN-γ if the concentration of allicin is augmented [38]. Moreover, it is well established that the presence of bacterial lipopolysaccharides and/or pro-inflammatory cytokines as IFN-γ o TNF-α in the host tissue stimulates the activation of M1-macrophages, implicated in both the inflammatory and the antimicrobial response [40]. Meanwhile, the M2-macrophages are activated in the presence of anti-inflammatory cytokines such as IL-10, IL-4 or IL-13 and are involved in tissue remodeling and repair processes [41]. The lower percentage of RAM-11 positive cells found in the PP + allicin-CHX group could correlate to a decrease in the M1-subpopulation, which would reduce the antimicrobial response of the activated macrophages. However, since the RAM-11 monoclonal antibody recognizes the total macrophage population, we cannot confirm this hypothesis. The combination of cytokine release studies and the quantification of M1- and M2- activated macrophages would allow us to further evaluate the effects of allicin and CHX on the macrophage activity.

It has been suggested that allicin should not be extensively utilized as an antibiotic because the dosages required for its administration would exert similar toxic side effects over the host cells and the bacteria [42]. Considering all these facts, we could assume that allicin interferes with the inflammatory processes and the macrophage response in the allicin-CHX treated implants and thereby favoring the survival of bacteria in the host tissue. On the contrary, mesh dipping in CHX could be effective to pretreat hernia mesh material prior to the implantation, which also occurs with other medical devices such as vascular catheters [29]. This solution contains 0.05% CHX, a much lower concentration than the 2–4% CHX utilized for disinfecting the surgical area [43] or the concentration present in the dermal patches for removal of catheters, which usually contains up to 20% CHX [44]. Because solutions containing CHX can be toxic for the patient [45], low concentrations are most likely to reduce the side effects of the treatment.

It is important to highlight the scarce information available on the interaction between allicin and implantable medical devices. To the best of our knowledge, in a setting of bacterial infection there is only one *in vivo* study involving allicin and biomaterials [46]. Since the antibacterial effectiveness of allicin is well demonstrated *in vitro* [30], further studies are needed to better understand the interaction between allicin and biomaterials as well as the performance of allicin-coated implants. Similarly, the development of diffusion studies using animal models would give us valuable information regarding the systemic toxicity of this antiseptic.

The main limitation of the present work is the absence of non-soaked PP implants utilized as negative control of the CHX and allicin-CHX treatments. Our previous experience with experimental models of infection caused by *Sa* [26,47] show that the animals undergoing non-coated implants develop strong wound infection, sometimes close to the endpoint indicators and even death. In such these situations, the welfare of the animals with non-coated implants is hampered, and the use of several animals providing scarce and not relevant data is not justified since does not accomplish with the 3R´s criteria (Replacement, Reduction, Refinement) that we must comply with.

To conclude, our results demonstrate that pretreating PP hernia repair material with a low concentration of CHX confers adequate antibacterial activity to the mesh without disturbing the short-time tissue repair and remodeling processes. Despite the positive results, the activity of the antiseptic could be gradually lost due to a rapid dilution of the agent into the host tissue [48], thus constituting a potential limitation of this approach upon translation to the clinic. More sophisticated antibacterial devices such as meshes provided with drug-loaded polymeric coatings can be developed [49], enhancing the effectiveness of the device. We therefore consider it necessary to continue with the study by designing a mesh with a polymer coating system that allows the local and controlled releasing of the agent without provoking any undesired systemic effects or hampering the tissue integration of the implant.
